# Correlation between overall survival and differential plasma and tissue tumor marker expression in nasopharyngeal carcinoma patients with different sites of organ metastasis

**DOI:** 10.18632/oncotarget.10676

**Published:** 2016-07-18

**Authors:** Hung-Ming Wang, Tung-Liang Lin, Yung-Chia Kuo, Hsin-Pai Li, Kai-Ping Chang, Chien-Yu Lin, Hsien-Chi Fan, An-Chi Lin, Chia-Hsun Hsieh, Ngan-Ming Tsang, Li-Yu Lee, Sheng-Chieh Chan, Kar-Wai Lui, Yu-Sun Chang, Cheng-Lung Hsu

**Affiliations:** ^1^ Division of Hematology-Oncology, Department of Internal Medicine, Chang Gung Memorial Hospital, Chang Gung University, Taoyuan, Taiwan, ROC; ^2^ Department of Cell and Molecular Biology, Chang Gung University, Taoyuan, Taiwan, ROC; ^3^ Department of Otolaryngology-Head and Neck Surgery, Chang Gung Memorial Hospital, Chang Gung University, Taoyuan, Taiwan, ROC; ^4^ Department of Radiation Oncology, Chang Gung Memorial Hospital, Chang Gung University, Taoyuan, Taiwan, ROC; ^5^ Department of Pathology, Chang Gung Memorial Hospital, Chang Gung University, Taoyuan, Taiwan, ROC; ^6^ Department of Nuclear Medicine, Chang Gung Memorial Hospital, Chang Gung University, Taoyuan, Taiwan, ROC; ^7^ Department of Diagnostic Radiology, Chang Gung Memorial Hospital, Chang Gung University, Taoyuan, Taiwan, ROC

**Keywords:** nasopharyngeal carcinoma, metastasis, EB virus, EBV DNA, IP-10

## Abstract

Differential overall survival of nasopharyngeal carcinoma (NPC) with different organ site metastases has been documented. Here, we attempted to determine the underlying mechanisms by assessing plasma and tumor tissue markers in relation to patient survival. Pretreatment plasma Epstein-Barr virus (EBV) DNA concentrations, cytokines and tissue macrophages, proliferation and apoptosis markers were determined in 178 patients with metastatic NPC. The median overall survival (OS) was 19 months. Patients with single organ metastases had better outcomes than those with multiple organ metastases (median OS: 26 months vs. 16 months), with statistical significance. Among the single organ involvement cases, patients with lung metastasis only showed longer survival than those with bone or liver involvement (median OS: 50 months vs. 21 months vs. 18 months; *P* < 0.001). Pretreatment plasma EBV DNA concentrations were lower in patients with lung metastasis than bone or liver metastasis among single organ site groups. Plasma interferon-γ-inducible protein-10 (IP-10) and monocyte chemotactic protein-1 (MCP-1) expression levels were correlated with differential single organ site metastasis OS and EBV DNA load. Liver metastatic tissue had higher density of infiltrating macrophages and proliferative index than the lung metastatic group. Low pretreatment plasma EBV DNA load, expression of cytokines, such as IP-10 and MCP-1, tissue macrophage infiltration, and proliferative index may contribute to the differences in overall survival.

## INTRODUCTION

Nasopharyngeal carcinoma (NPC) is a commonly diagnosed disease in southeastern Asia. Improvements in diagnostic techniques, including use of positron emission tomography-computed tomography (PET-CT) scanning, have facilitated early detection of distant metastases (DM) in patients [1]. Advances in local therapy, including intensity-modulated radiotherapy (IMRT) and concurrent chemoradiotherapy (CCRT), have led to the identification of DM as a major cause of treatment failure [2–4]. Outcomes of salvage treatment are currently poor and disease content at the time of recurrence plays a pivotal role in patient survival.

The spectrum of DM in NPC is heterogeneous and survival durations are variable. Aggressive multimodal therapy may facilitate long-term patient survival [5–7]. Distinct outcomes have been reported in patients with DM in different organs. For example, patients with lung metastases have a good prognosis and longer survival [6, 8], whereas liver metastases are associated with dismal prognosis and shorter survival [9]. Better outcomes have been documented for patients with solitary nodule metastases than those with multiple nodules/sites of metastases [7]. However, the underlying reasons for differences in outcomes are yet to be established.

NPC is an Epstein-Barr virus (EBV)-associated disease, whereby EBV plays a pivotal role in tumor initiation and progression [10]. The plasma EBV DNA concentration is an efficient tumor indicator with high sensitivity and specificity in patients with NPC [11, 12]. Plasma EBV DNA load is significantly correlated with tumor burden and has been incorporated into the TNM staging system [13]. Additionally, plasma EBV DNA load is a more sensitive marker of DM than local recurrence in patients with NPC, with a higher rate of positivity and viral load [14]. In NPC patients with DM, lower viral load before treatment and shorter viral clearance time are associated with better outcomes, and complete eradication of EBV leads to longer overall survival [15, 16].

Chronic inflammation is related to the development of various cancer types [17] and enriched presence of inflammatory cells and mediators associated with poor prognosis due to metastasis [18, 19]. Chemokines and their corresponding receptors play a major role in cancer-associated inflammation and affect the composition and function of cells within the tumor microenvironment [19, 20]. Inflammatory chemokines, such as monocyte chemoattraction protein 1/chemokine C-C motif ligand 2 (MCP-1/CCL2) and CCL5, may function in recruitment of cells during inflammation. Homeostatic chemokines, including chemokine C-X-C motif ligand 12 (CXCL12) and CCL19, are constitutively expressed in specific tissues and predominantly regulate homeostatic trafficking of leukocytes [21, 22]. Elevated levels of CCL2 and/or CCL5 are associated with poor outcomes due to the high incidence of metastasis in various types of cancer [23–25].

## RESULTS

### Patients, treatments, and outcomes

From January 2007 to January 2016, we prospectively enrolled a total of 178 patients with metastasis, including 56 newly diagnosed with DM and 122 with recurrent DM [16]. PET-CT scans were used to evaluate 151 of the 178 patients (84.8%), including 52/56 (92.9%) newly diagnosed and 99/122 (81.1%) with recurrent DM. All examined patients were positive for DM. Patient characteristics are shown in Table 1. Among the 178 patients, 137 (77.0%) had good performance status (ECOG 0-1) and 151 (84.8%) had undifferentiated carcinoma cell type. Ten of the 56 (17.9%) newly diagnosed patients and 59 of the 122 (48.4%) with recurrent disease were pathologically confirmed as metastatic. Moreover, 38 of the 122 (31.1%) patients displayed local regional recurrence in addition to DM. Cisplatin-based chemotherapy regimens including gemcitabine plus cisplatin (G + P) and PUL-based regimen of cisplatin (P) + oral tegaful + uracil (UFT) + oral calcium folinate (L) combined with mitomycin-c (M) or bleomycin (B) were the major treatment option for these patients [16, 26]. Forty-six patients received local therapy, including irradiation boost, CCRT or surgery, on primary or metastatic lesions.

**Table 1:** Relationships of treatment response and overall survival with baseline demographic and clinical characteristics in 178 NPC patients with DM

Parameters	Treatment Response			Overall Survival			
	No.(%)	No. of Responders (%)	*P*	2-y rate (%)	Median (months)	HR (95% CI)	*P*
**Age (y)**							
≤50	86 (48.3)	43 (50.0)	0.764	40.7	20	0.894 (0.649–1.231)	0.483
> 50	92 (51.7)	49 (53.3)		33.7	18		
**Sex**							
Male	149 (83.7)	75 (50.3)	0.426	37.6	19	1.140 (0.742–1.752)	0.543
Female	29 (16.3)	17 (58.6)		44.8	21		
**Performance status (ECOG)**							
≤1	137 (77.0)	78 (56.9)	0.013	46.7	23	0.410 (0.283–0.593)	< 0.001
> 1	41 (23.0)	14 (34.1)		9.8	10		
**Pathology**							
Undifferentiated carcinoma	151 (84.8)	78 (51.7)	1.000	39.7	20	0.935 (0.600–1.458)	0.763
Nonkeratinizing carcinoma +Squamous cell carcinoma	27 (15.2)	14 (51.9)		33.3	18		
**Disease-free interval**							
≤6 months	69 (38.8)	37 (40.2)	0.759	29.0	15	1.399 (1.011–1.938)	0.038
> 6 months	109 (61.2)	55 (59.8)		45.0	22		
**Chemotherapy Regimen**							
G + P	57 (32.0)	36 (63.2)	0.038	49.1	23	0.771 (0.546–1.089)	0.131
PUL(B/M)	121 (68.0)	56 (46.3)		34.7	17		

* The 2002 American Join Committee on Cancer staging system was used.

Responder: complete response (41) + partial response (51); G+P: gemcitabine + cisplatin; PUL(B/M): cisplatin + UFT + calcium folinate + (bleomycin or mitomycin-c).

Evaluation of treatment response after 3 months disclosed that 41 patients (23.0%) achieved complete response (CR), 51 (28.7%) achieved partial response (PR), and 23 (12.9%) had stable disease (SD). In chemotherapy group analysis, although G + P had better response than PUL(B/M) (*P* = 0.038) but overall survival did not reach statistical significance (*P* = 0.131). As of February 2016, 151 (84.8%) patients had died, 2 (1.1%) were alive with disease, and 25 (14.1%) were alive without disease. Median follow-up duration for surviving patients was 57 months (range, 25–117 months).

### Plasma EBV DNA quantitation assay

**Figure 1:**
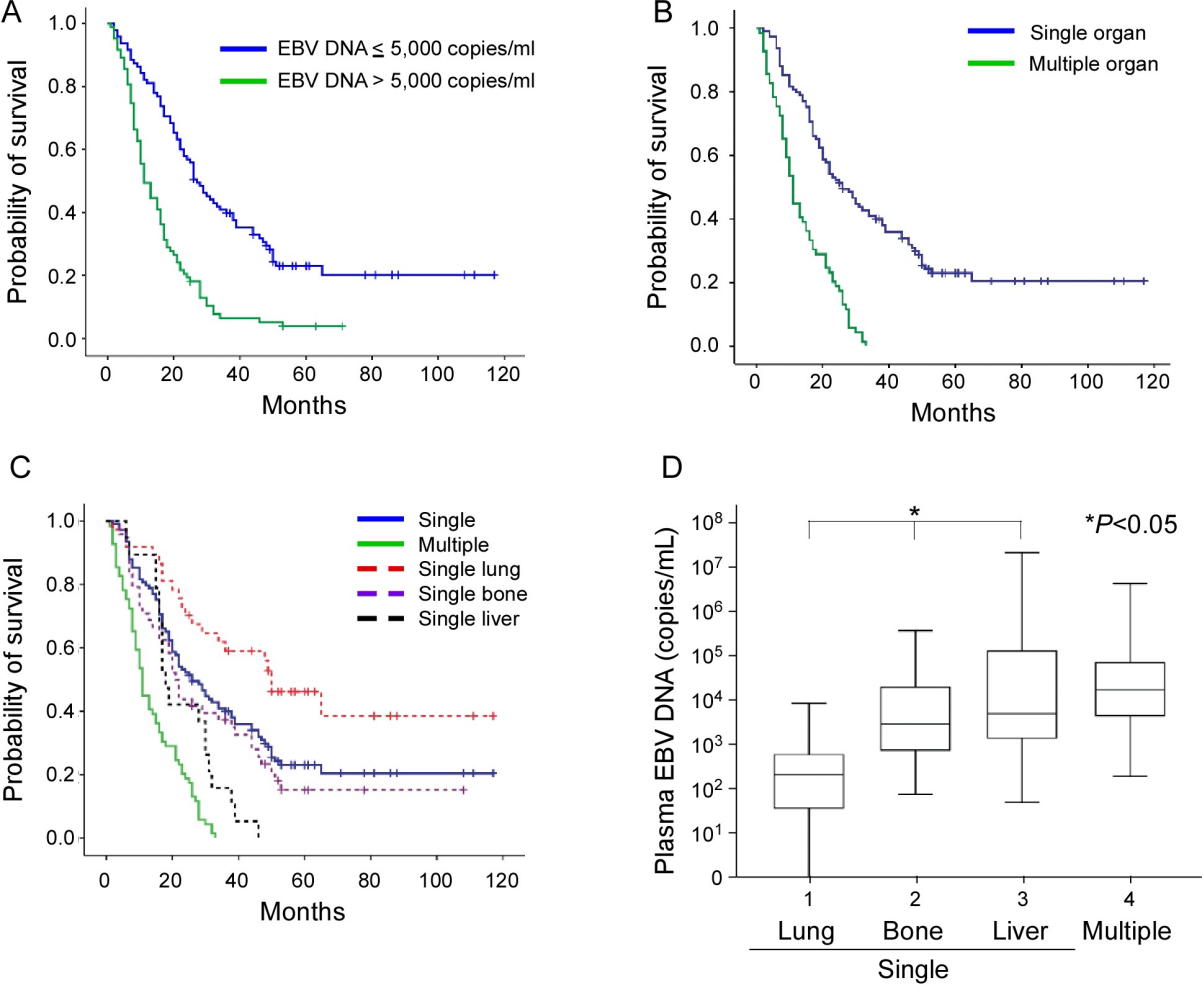
Kaplan-Meier estimate of overall survival according to (**A**) pretreatment EBV DNA concentration (≤ 5,000 versus > 5,000 copies/mL), (**B**) number of organ metastases (single versus multiple sites), and (**C**) single versus multiple organ metastasis and subsite analysis of single organ sites (lung, bone or liver). (**D**) Box plot of pretreatment EBV DNA concentrations in NPC patients with metastases, including single organ metastasis of lung, bone and liver, and multiple organ metastases. The EBV DNA concentration was expressed as a natural log scale.

Blood samples were obtained from each patient for EBV DNA quantitation analysis before chemotherapy. Pretreatment plasma EBV DNA concentrations ranged from 0 to 20,762,916 copies/mL (median, 4,220 copies/mL; mean, 303,894 copies/mL). Median OS for all patients with DM was 19 months. Using a pretreatment EBV DNA load of 5,000 copies/mL as a cutoff point established previously ([16]), among the 178 patients, 95 (53.4%) had ≤ 5,000 copies/mL, including 11 with undetectable EBV DNA, and 83 (46.6%) had > 5,000 copies/mL EBV DNA. Median OS in these two groups was 27 months (95% CI, 21.8–32.2 months) and 11 months (95% CI, 8.0–14.0 months), respectively (*P* < 0.001, HR: 2.602, 95% CI, 1.868–3.623) (Table 2, Figure 1A), consistent with our previous report [16] and other studies [15].

**Table 2:** Metastatic sites, EBV DNA load and overall survival in 178 NPC patients with DM

	No.	EBV DNA (copies/mL)			Overall Survival (OS)		
		≤ 5,000 No.(%)	> 5,000 No.(%)	*P* value	OS (months) (95% CI)	*P* value	HR (95% CI)
EBV DNA, copies/mL							
ALL					19.0 (15.7–22.3)		
≤ 5,000	95				27.0 (21.8–32.2)	< 0.001	1.000
> 5,000	83				11.0 (8.0–14.0)		2.602 (1.868 – 3.623)
Organ site metastasis							
Single vs. multiple					19.0 (15.7–22.3)	< 0.001	
single	109	75	34	< 0.001	26.0 (19.5–32.5)		1.000
multiple	69	20	49		16.0 (13.5–18.4)		3.395 (2.380 – 4.843)
Single organ site metastasis§				< 0.001	28.0 (21.0–35.0)	< 0.001	
Lung	37	35	2	50.0 (33.7–66.3)			1.000
Bone	48	28	20	21.0 (18.1–23.9)			2.310(1.341 – 3.979)
Liver	19	9	10	18.0 (15.4–20.6)			3.710(1.926 – 7.149)

§ does not include metastasis to lymph nodes or soft tissue in the other 5 patients.

### Analysis of metastatic organ number in relation to OS

Median OS in the single organ site metastasis group was 26 months (95% CI, 19.5–32.5 months), which was longer than that recorded for multiple organ site metastases (16 months, 95% CI, 13.5–18.4 months), as shown in Table 2 and Figure 1B.

Previous investigations have revealed better outcomes of lung metastasis [6, 8] and poorer overall survival of liver metastasis patients [9]. To further validate these findings, we analyzed patients with single organ metastasis at the most frequent sites (lung, bone, and liver). In total, 109 patients were diagnosed with single organ site metastasis, including 37 lung, 48 bone, 17 liver, and 5 lymph nodes or soft tissue. In subgroup analysis, the lung metastasis group had longest OS of 50 months (95% CI, 33.7–66.3 months), compared to the bone group (21 months; 95% CI, 18.1–23.9 months) and liver group (18 months; 95% CI, 15.4–20.6 months), as presented in Table 2 and Figure 1C. Moreover, the lowest pretreatment EBV DNA concentration was recorded in the lung metastasis group (median 216 copies/mL, mean 902 copies/mL), followed by bone metastasis (median 2,965 copies/mL, mean 21,587 copies/mL), and liver metastasis (median 5,046 copies/mL, mean 1,907,519 copies/mL) groups, as shown in Figure 1D. In terms of cutoff value, the lung metastasis group with < 5,000 copies/ml EBV DNA contained more patients (35/37; 94.6%), compared to bone (28/48; 58.3%) and liver metastasis groups (9/19; 47.4%), with statistical significance (Table 2).

### Multivariate analysis

Multivariate Cox proportional hazard analysis was performed with *P* values < 0.05 taken as significant in univariate analysis. Performance status (HR, 0.451; 95% CI, 0.310–0.656; *P* < 0.001), pretreatment EBV DNA concentration (HR, 1.941; 95% CI, 1.330–2.832; *P* = 0.001), and metastatic organ site number (HR, 2.498; 95% CI, 1.710–3.649; *P* < 0.001) remained significant predictors of OS (Table 3), but not disease-free interval (HR, 0.968; 95% CI, 0.683–1.373; *P* = 0.856), as shown in Table 3.

**Table 3:** Multivariate analysis

	*P*	HR	95% of CI	
			Lower	Upper
Performance status	< 0.001	0.451	0.310	0.656
Disease free interval	0.856	0.968	0.683	1.373
EBV DNA	0.001	1.941	1.330	2.832
Single organ or multiple organ site metastasis	< 0.001	2.498	1.710	3.649

### Plasma cytokines involved in OS of NPC with different organ site metastases

**Figure 2:**
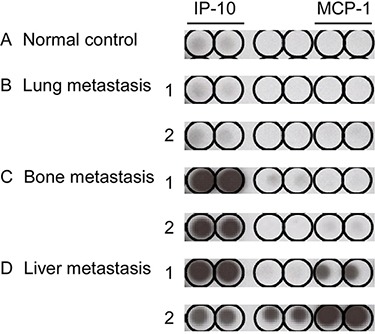
Cytokine array of pretreatment plasma in different sites of single organ metastasis Two samples from each single organ site metastatic group were selected: (**B**) lung, (**C**) bone, and (**D**) liver. One normal healthy sample served as the control (**A**) for cytokine analysis. Tests for each cytokine were performed in duplicate.

**Figure 3:**
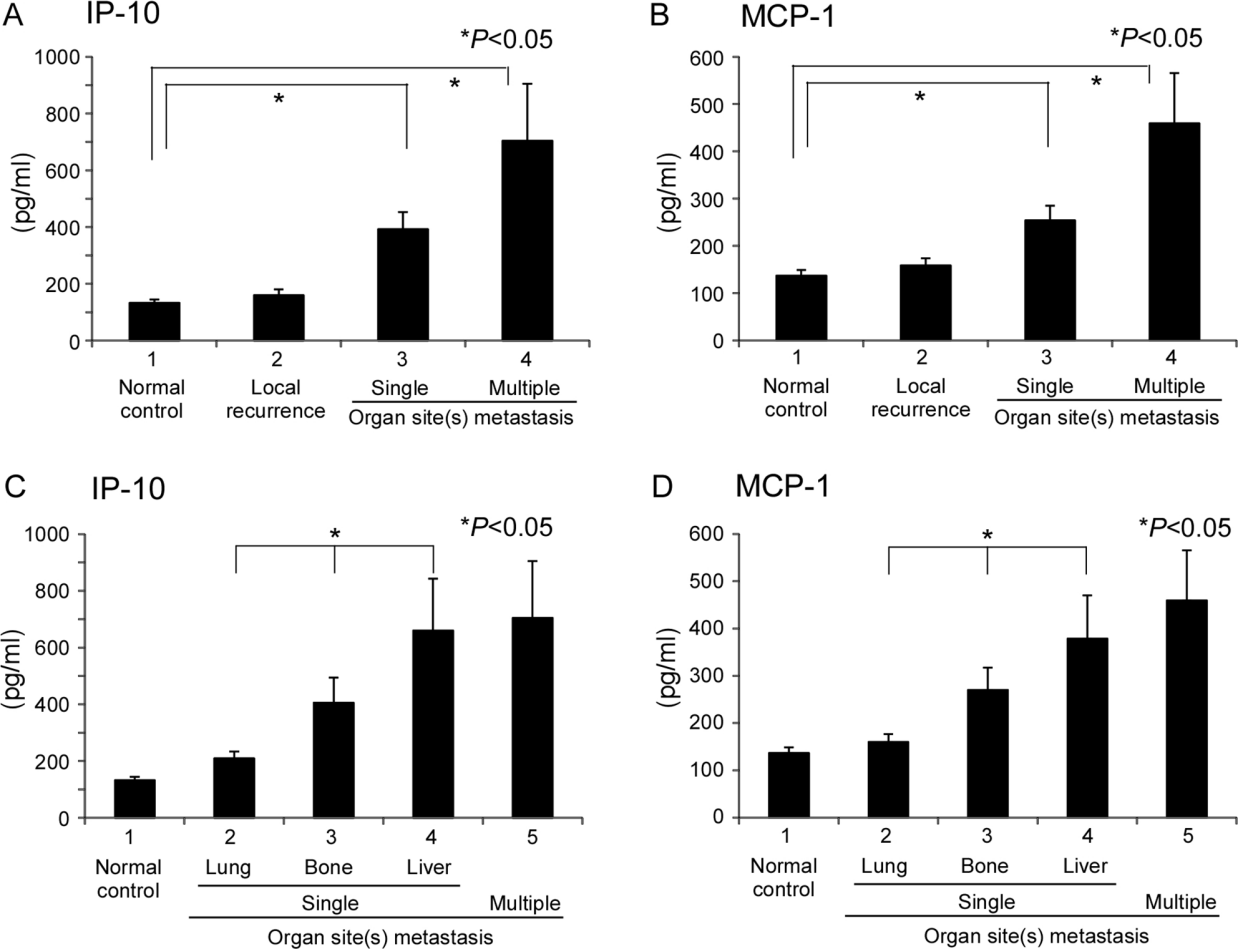
Quantitation of candidate cytokines in single or multiple organ site metastases ELISA of plasma samples from single or multiple organ sites for (**A**) IP-10 and (**B**) MCP-1. Samples from normal healthy volunteers and local recurrence patients served as the control. Subsite analysis of single organ site metastasis, specifically, lung, bone, and liver, for (**C**) IP-10 and (**D**) MCP-1.

In NPC, the EB virus is closely related to disease progression and inflammation/immunologic response of the host. We were interested in determining whether the inflammation/immunologic response is associated with differential organ site metastasis outcomes. Accordingly, plasma cytokine array of the three single organ site metastasis groups was performed before treatment. As shown in Figure 2, interferon-γ-inducible protein-10 (IP-10) and MCP-1 were differentially expressed among the three groups. ELISA was performed to further validate these results. In the IP-10 assay, a mean value of 392.8 pg/ml was obtained for the single organ site metastatic group that was higher than that of the normal control (133.5 pg/ml) and local recurrence (160.2 pg/ml) groups but lower than the multiple organ site metastasis group (705.0 pg/ml), with statistical significance. Upon further separation of the single organ site metastasis groups into lung, bone, and liver subgroups in IP-10 ELISA analysis, the lung group had a significantly lower mean value (209.9 pg/ml), compared to the bone group (406.2 pg/ml) and liver group (660.2 pg/ml) (Table 4, Figure 3A and 3C). The mean value of MCP-1 was higher in the single organ site metastasis group (254.0 pg/ml) than normal control (137.3 pg/ml) and local recurrence (159.1 pg/ml) groups, but lower than the multiple organ site metastasis group (459.8 pg/ml). Subgroup analysis of single organ sites (lung, bone, and liver) revealed a similar pattern to IP-10, with decreasing mean values as follows: liver (378.8 pg/ml) > bone (270.6 pg/ml) > lung (160.7 pg/ml) (Table 4, Figure 3B and 3D).

**Table 4:** Valid candidate cytokine (IP-10 and MCP-1) levels in different organ sites of metastasis

	No.	IP-10	MCP1
Normal control	28	133.5 ± 11.4	137.3 ± 11.5
Local recurrence	20	160.2 ± 20.2	159.1 ± 14.6
Metastasis (organ site(s))	173		
Single	104	392.8 ± 59.9	254.0 ± 30.6
Lung	37	209.9 ± 23.9	160.7 ± 16.0
Bone	48	406.2 ± 87.8	270.6 ± 46.8
Liver	19	660.2 ± 182.6	378.8 ± 91.2
Multiple	69	705.0 ± 199.7	459.8 ± 105.6

Expressed as mean ± standard error of mean.

For IP-10, single vs. multiple: *P* = 0.052; single organ metastasis: lung vs. bone vs. liver: *P = 0.009; lung vs. liver: *P = 0.024.

For MCP-1, single vs. multiple: *P* = 0.078; in single: lung vs. bone vs. liver: *P = 0.013; lung vs. liver: *P = 0.029.

### Correlation of differential cytokine expression at different sites of single organ metastasis with EBV DNA load

**Figure 4:**
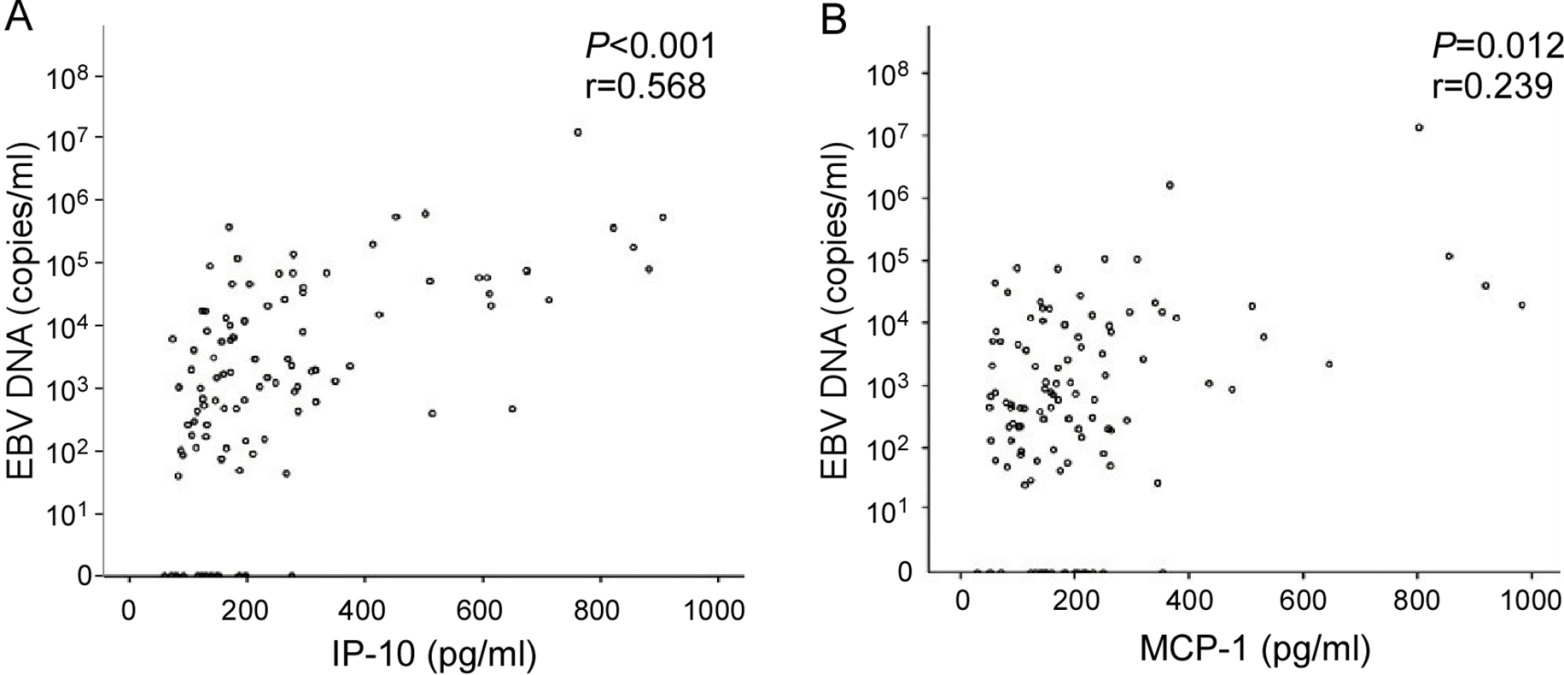
Correlation between plasma levels of cytokine markers and EBV DNA load Spearman's correlation analysis between cytokine markers for NPC and EBV DNA load. (**A**) IP-10, (**B**) MCP-1.

Since IP-10 and MCP-1 displayed similar quantitative distribution patterns in single organ site metastasis (i.e., lower levels in lung than bone and liver metastases), we were interested in determining whether expression patterns of these cytokines are associated with the EBV DNA load. Spearman's correlation analysis between EBV DNA load and concentration of analytes revealed that both IP-10 and MCP-1 are significantly correlated with EBV DNA load of local recurrence and distant metastatic patients (*r* = 0.568, *P* < 0.001; *r* = 0.239, *P* = 0.012, respectively) (Figure 4).

### Higher infiltration of macrophages in metastatic liver than lung tumors

**Figure 5:**
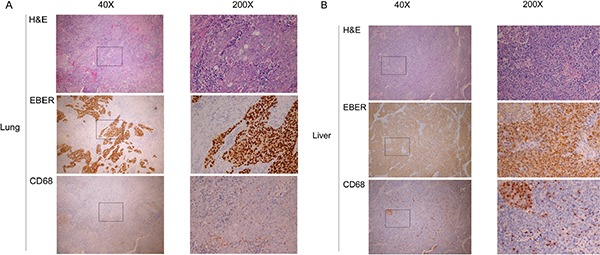
Macrophage infiltration in different sites of single organ metastasis H&E staining, EBER staining for EBV-positive cells, and anti-CD68 staining for macrophages at low power (40×) and high power (200×) fields for (**A**) lung and (**B**) liver.

MCP-1 is a macrophage chemoattractant protein, and high density of macrophage infiltration has been reported in tumors with poor prognosis. Examination of metastatic tumor tissues from different organ sites using anti-macrophage immunohistochemical staining showed that metastatic liver tumors have higher infiltrating macrophage density than metastatic lung tumors (Figure 5).

### Tissue markers contribute to variable OS in NPC with differential organ site metastases

**Figure 6:**
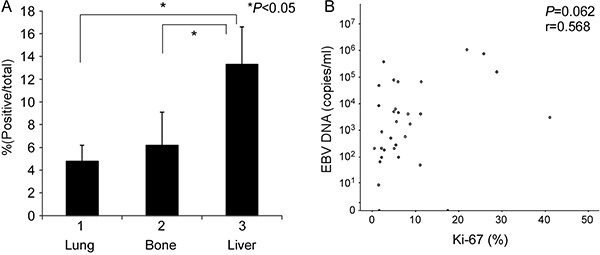
Determination of the proliferation index in different single organ site metastases (**A**) Ki-67 expression served as the proliferation index, assessed based on anti-Ki-67 immunochemical staining. (**B**) Spearman's correlation analysis between Ki-67 and EBV DNA load.

**Figure 7:**
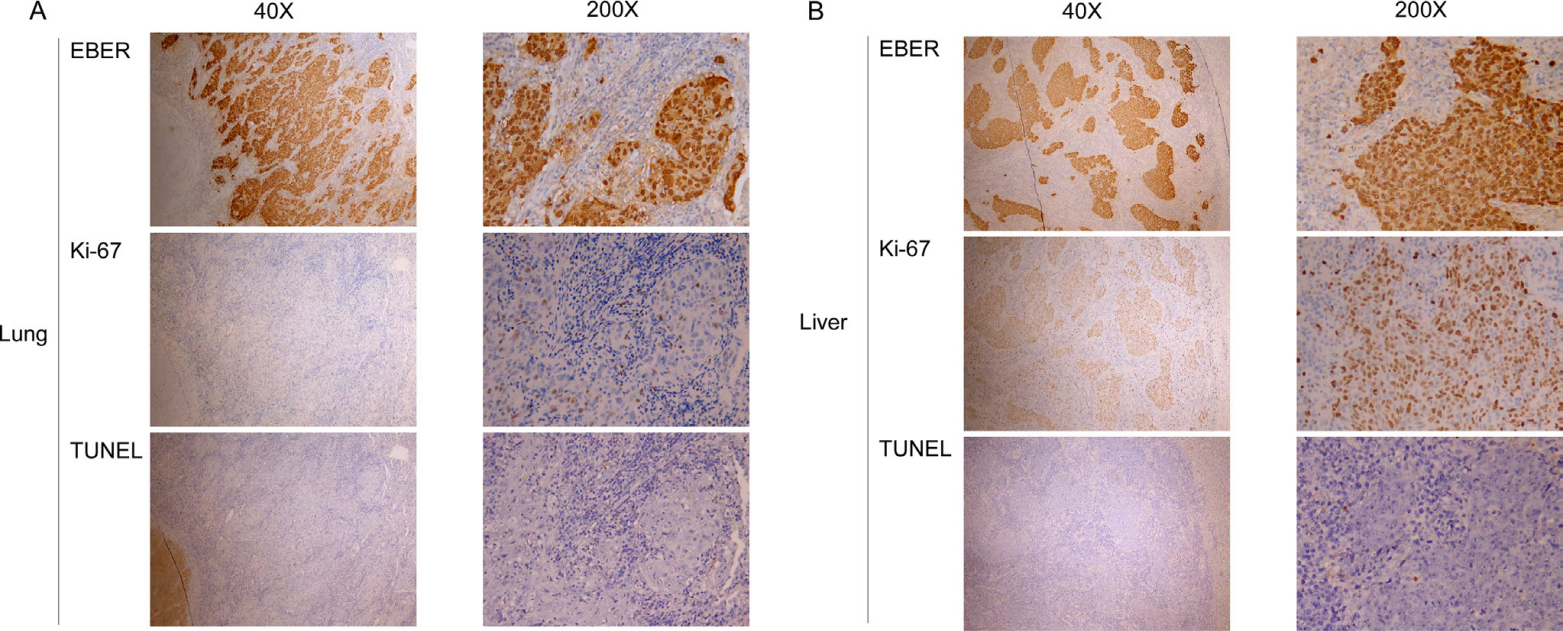
Proliferation and apoptotic indices in different sites of single organ metastases EBER staining for EBV-positive cells, proliferation index (anti-Ki-67 staining), and apoptotic index (TUNEL assay) at low power (40×) and high power (200×) fields for (**A**) lung and (**B**) liver.

In addition to the potential plasma factors contributing to differential organ site metastasis, we analyzed proliferation and apoptosis indices in the different metastatic sites. Specifically, Ki-67 protein expression was assessed via immunohistochemical staining for proliferation and the TUNEL assay performed for apoptosis analysis. Liver metastasis tumors had a significantly higher number of Ki-67-positive cells (mean ± standard error of mean, 13.3 ± 3.3%) than bone metastasis (6.2 ± 2.9%) and lung metastasis (4.8 ± 1.4%) tumors, as shown in Table 5 and Figure 6. Spearman's correlation analysis between EBV DNA load and positive rate of Ki-67 expression revealed the tendency of statistical significance (*r* = 0.568, *P* = 0.062, respectively)(Figure 6B). Consistently, Ki-67 staining was stronger in liver than lung metastasis cases (Figure 7). The TUNEL apoptotic assay disclosed no significant differences between lung and liver metastasis tumors, as shown in Figure 7.

**Table 5:** Proliferation index (Ki-67) in different single metastatic organs

Metastatic organ	No.	Ki-67
Lung	12	4.8 ± 1.4
Bone	8	6.2 ± 2.9
Liver	12	13.3 ± 3.3

Expressed as mean ± standard error of mean.

Three groups (non-parametric test), *P = 0.020.

Lung vs. liver: *P = 0.018; Bone vs. liver: *P*= 0.247.

## DISCUSSION

Consistent with previous findings, we confirmed that independent significant factors predictive of reduced survival include poor performance status, high plasma EBV load and multiple organ involvement whereas single organ metastasis is a factor predictive of increased OS [6, 9, 27]. Within the single organ metastasis groups, median OS was longer in patients with lung (50 months) metastasis than those with bone (21 months) or liver (18 months) metastasis. Among the 37 patients with lung metastasis only, 18 (48.6%) had solitary nodules and 35 (94.6%) contained pretreatment EBV DNA load ≤ 5,000 copies/mL. Patients with lung metastasis additionally displayed a better treatment response (28/37 (76.7%), 21 CR + 7 PR) than those with only bone (29/48 (60.4%), 10 CR + 19 PR) or liver (11/19 (57.9%), 4 CR + 7 PR) metastases. In our 37 patients with lung metastasis, 12 (32.4%) received local therapy, including surgery in 8 and CCRT in 4, along with systemic chemotherapy before and/or after local therapy.

Lower pretreatment EBV DNA concentration is a significant predictor of better treatment response and longer OS [15, 16]. Consistent with earlier findings, pretreatment EBV DNA was lower in patients with lung metastases, compared to groups of patients with bone or liver metastases only. Furthermore, liver involvement was evident in all 12 patients with EBV DNA load > 400,000 copies/mL, potentially explaining the poor outcomes associated with liver metastasis. In cytokine studies, patients in the lung metastasis group had lower IP-10 and MCP-1 levels than those in the bone and liver groups. Proliferation index assessment showed lower levels of the proliferation marker, Ki-67, in the lung metastasis group, relative to those in patients with liver metastasis alone. Longer disease-free interval, lower pretreatment EBV DNA load, more solitary metastatic lesions, higher therapeutic response, lower cytokine expression, low proliferation index and longer OS signified a more favorable and indolent nature of the lung metastatic subgroup of NPC.

IP-10 is secreted from a variety of (mainly inflammatory) cells in response to interferon-γ stimulation [28]. This cytokine is also a chemoattractant for monocytes, T cells and NK cells, binds the CXCR3 receptor to exert its biological effects, and is involved in apoptosis, cell proliferation, and angiostatic effects [29]. In pancreatic cancer, IP-10 induction in stroma cell could recruit CXCR3+ T lymphocyte, lead to immunosuppressive and tumor-promoting effects, and correlate with poor survival [30]. Earlier studies revealed that IL-6, IL-8, IP-10, and MIP-3α are correlated with EBV DNA load in advanced NPC patients [31, 32]. In the current study, IP-10 was differentially expressed in patients with NPC displaying different organ metastases, with high levels in bone, liver, and multiple organs leading to poor prognosis and low levels in lung metastasis cases with longer survival.

MCP-1 may exert effects on tumorigenesis and metastasis via direct tumor growth stimulation or indirect microenvironment regulation through modulation of macrophage function [33]. High levels of MCP-1 and CCR2 are significantly associated with NPC metastasis and poor overall survival [34]. The MCP-1-CCR2 axis is suggested to activate extracellular signal-regulated kinase (ERK1/2) and matrix metalloproteinase (MMP) 2 and 9 pathways. The MCP-1 -2518(A/G) single nucleotide polymorphism has been shown to be a valuable genetic marker for distant metastasis risk evaluation in NPC patients after radiotherapy [35]. In many cancer types, MCP-1 expression is associated with increased infiltration of tumor-associated macrophages critical for tumor progression, growth and angiogenesis [23]. Data from the current study showed differential expression of MCP-1 in NPC patients with higher levels in liver than lung metastasis. Furthermore, in liver metastasis samples, we observed greater macrophage infiltration, potentially in response to higher levels of plasma MCP-1, compared to lung metastasis.

Ki-67, a proliferative marker, is highly expressed in NPC primary tissue and positively correlated with nodal involvement and tumor stage to a significant extent [36]. In the current study, we observed a high proliferation index (Ki-67) but low apoptotic index (TUNEL assay) in pure liver metastasis, compared to lung metastasis with low levels of both indices. Our data are consistent with the finding that EBV DNA load and IP-10 and MCP-1 cytokine expression contribute to variable overall survival in NPC with differential organ site metastases.

## MATERIALS AND METHODS

### Patients and treatment

Patients with biopsy-proven NPC and measurable metastatic disease were included, and the metastatic site(s) identified via examination of tissue samples or at least two imaging modalities. All patients had ECOG performance status ≤ 3, total bilirubin < 2.0 mg/dL, serum creatinine < 2.0 mg/dL, white blood cell count > 3,000/μL and platelet count > 75,000/μL. The cause of death was not locoregional disease. Twenty-eight patients with local recurrence and 20 control volunteers were enrolled in cytokine studies. This study was approved by the Institutional Review Board of Chang Gung Memorial Hospital, and all participants provided written informed consent. All patients were treated in our outpatient department using a protocol approved by the Nasopharyngeal Cancer Team of Chang Gung Memorial Hospital, Linkou. First-line chemotherapy in patients with metastatic disease was cisplatin-based until disease progression [16]. The second-line chemotherapy regimen was at the discretion of individual physicians. Local treatment, including surgery, irradiation, and CCRT to local/metastatic sites, was recommended where clinically applicable. Treatment response was evaluated every 3 months according to WHO criteria.

### DNA Extraction from plasma and real-time quantitative polymerase chain reaction (Q-PCR)

DNA was extracted from plasma as described previously [11, 16]. Briefly, 10 mL samples of peripheral blood were collected in an EDTA-treated tube and centrifuged at 1,000 × *g* for 15 min. Plasma DNA was extracted with a QIAamp DNA Blood MiniKit (Qiagen). About 500 to 1,000 μL of each sample per column (supplied in the QIAamp kit) was used for DNA extraction. DNA was eluted from each column with 80 μl distilled water [12]. EBV DNA concentrations were measured with real-time quantitative PCR of the *Bam*HI-W region of the EBV genome [11]. Primer and probe sequences, including the dual fluorescence-labeled oligomer, and the detailed procedures are described in a previous report [16].

### Cytokine array

Cytokine Arrays (kit ARY005, R&D systems, Minneapolis, USA) were performed using human plasma samples collected from peripheral blood after centrifugation at 1,000 × *g* for 30 min in an EDTA tube. The plasma sample was pre-mixed with a detection antibody cocktail and applied to the blocked array membrane. Signals from different plasma samples reacting with membranes within the same time were detected simultaneously. Arrays were conducted according to the manufacturer's instructions, and allowed the parallel determination of 36 cytokines. In this study, D5-D10 immunoblots were adapted from original membrane spots.

### Enzyme-linked immunosorbent assay (ELISA) of cytokines

Human plasma CXCL10/IP-10 and CCL2/MCP-1 levels were measured using the Quantikine Human IP-10 and MCP-1 kits from R&D Systems (Minneapolis, USA), according to the manufacturer's instructions. Plasma samples were collected in a similar manner as for cytokine array, and IP-10 or MCP-1 standards and plasma samples added to the ELISA plate pre-rehydrated with Assay Diluent. After incubation at room temperature for 2 h, ELISA plates were washed, incubated with IP-10 or MCP-1 conjugates for 2 h, and re-washed. Substrate Solution was added to the plate for 30 min at room temperature, followed by Stop Solution. Finally, optical densities were determined with TECAN^®^ ELISA READER Infinite 200 (Männedorf, Switzerland).

### Immunohistochemical staining

Paraffin-embedded tumor sections (5 μm) were deparaffinized, rehydrated, and submerged in citrate buffer (pH 6.0) for antigen retrieval. After washing, nonspecific signals were blocked with Hydrogen Peroxide Block and Ultra V Block reagents. Sections were incubated with or without mouse anti-CD68 (clone KP1, DAKO IS609) or mouse anti-Ki-67 antibody (clone MIB-1, DAKO M7240, 1:100 dilution) for 90 min at room temperature. Finally, slides were stained with the UltraVision Quanto Detection System HRP DAB (Thermo Scientific, Waltham, MA) using the Primary Antibody Amplifier Quanto/HRP Polymer Quanto for signal amplification, DAB Quanto Chromogen/Substrate for signal visualization, and hematoxylin for counterstaining. The Ki-67 signal was analyzed using ImageScope software (Leica Biosystems).

### *In situ* TUNEL assay

Apoptosis was determined by detecting DNA fragmentation using the DeadEnd Colorimetric terminal deoxyribonucleotidyl transferase (TdT)–mediated biotin-16-dUTP nick end-labeling (TUNEL) System (Promega, Madison, WI) in accordance with the manufacturer's instructions. Briefly, 5 μm paraffin-embedded tumor sections were deparaffinized and rehydrated. After washing, slides were fixed in 10% buffered formalin in PBS for 15 min and subsequently treated with proteinase K (20 μg/mL) for 15 min at room temperature. Subsequently, slides were refixed and incubated with equilibration buffer for 10 min. After removal of equilibration buffer, sections were covered with TdT reaction mixture for 1 h. The reaction was terminated by immersing slides in 2 standard saline citrate (SSC) buffer for 15 min. Slides were treated with 0.3% hydrogen peroxide in PBS to block endogenous peroxidase activity. After washing, sections were incubated with streptavidin horseradish peroxidase (HRP) solution for 30 min at room temperature, developed with diaminobenzidine (DAB) solution and counterstained with Mayer's hematoxylin.

### Statistical analysis

Categorical data were compared using the χ^2^ test or Fisher's exact test, where appropriate. Continuous data were compared with the *t*-test. Overall survival (OS) was calculated from the beginning of chemotherapy to the date of death or last follow-up. The disease-free interval was calculated from the last day of previous treatment to the date of documented distant failure, with newly diagnosed DM defined as 0 month. Univariate comparison of survival was performed using the log-rank test. Multivariate analysis with Cox's proportional hazards model was used to estimate hazard ratio (HR) and 95% confidence interval (CI) [37]. Spearman's rank correlation was used to measure the correlation between the plasma cytokine levels and plasma EBV DNA load. All analyses were two-sided, and *P* values < 0.05 considered statistically significant. All statistical analyses were performed using SPSS (version 17.0.).
